# Transition of young adults with metabolic bone diseases to adult care

**DOI:** 10.3389/fendo.2023.1137976

**Published:** 2023-03-17

**Authors:** Jordan Ross, Michelle R. Bowden, Christine Yu, Alicia Diaz-Thomas

**Affiliations:** ^1^ Division of Pediatric Endocrinology, University of Tennessee Health Science Center, Memphis, TN, United States; ^2^ Division of General Pediatrics, University of Tennessee Health Science Center, Memphis, TN, United States; ^3^ Le Bonheur Children’s Hospital, Memphis, TN, United States; ^4^ Endocrinology Division, St. Jude Children’s Research Hospital, Memphis, TN, United States

**Keywords:** transition to adult care, metabolic bone disease, hypophosphatasia, X-linked hypophosphatemic rickets (XLH), osteogenesis imperfecta, delayed puberty, eating disorder (ED), cancer survivorship

## Abstract

As more accurate diagnostic tools and targeted therapies become increasingly available for pediatric metabolic bone diseases, affected children have a better prognosis and significantly longer lifespan. With this potential for fulfilling lives as adults comes the need for dedicated transition and intentional care of these patients as adults. Much work has gone into improving the transitions of medically fragile children into adulthood, encompassing endocrinologic conditions like type 1 diabetes mellitus and congenital adrenal hyperplasia. However, there are gaps in the literature regarding similar guidance concerning metabolic bone conditions. This article intends to provide a brief review of research and guidelines for transitions of care more generally, followed by a more detailed treatment of bone disorders specifically. Considerations for such transitions include final adult height, fertility, fetal risk, heritability, and access to appropriately identified specialists. A nutrient-dense diet, optimal mobility, and adequate vitamin D stores are protective factors for these conditions. Primary bone disorders include hypophosphatasia, X-linked hypophosphatemic rickets, and osteogenesis imperfecta. Metabolic bone disease can also develop secondarily as a sequela of such diverse exposures as hypogonadism, a history of eating disorder, and cancer treatment. This article synthesizes research by experts of these specific disorders to describe what is known in this field of transition medicine for metabolic bone diseases as well as unanswered questions. The long-term objective is to develop and implement strategies for successful transitions for all patients affected by these various conditions.

## 1 Introduction

Adolescence is a vulnerable period with many changes in a person’s interpersonal, vocational, and societal roles. Those without chronic medical conditions deserve continuous access to medical care from adult healthcare providers who can continue to provide preventive care throughout the lifespan. Adolescents who experience chronic medical conditions have the right to expect this same standard of medical care as adults, regardless of the complexity or rarity of a particular medical history. The American Academy of Pediatrics has provided an updated statement in support of this goal, including strategies for its realization (1).

As the responsibility for healthy transitions becomes the shared and equal effort of adult and pediatric healthcare teams, there is an opportunity to consider the persistent, evolving, and new aspects of a chronic disease that an adult may experience differently from a child (1). Evidence-based guidance, when available, is critical to build a productive management strategy for adults starting in the pediatric/adult health transition. Evidence-based guidelines and consensus statements from stakeholders in the medical community are needed so that adult providers who increasingly encounter survivors of pediatric medical conditions have a wealth of resources available. Included in this general vision is the application of transitions of care to primary endocrinologic conditions and other chronic conditions with endocrine aspects (e.g. cystic fibrosis-related diabetes, chronic glucocorticoid exposure in autoimmune and inflammatory diseases, a disrupted parathyroid axis in patients with DiGeorge syndrome).

It is important to consider the values and expectations of patients in designing transition programs and procedures (2). An endocrine transition program in Israel showed that patients expressed a high degree of satisfaction with such a program, particularly if these patients were able to adhere to follow-up in the respective adult clinic (3). Much work has gone into the transition of children with Type 1 diabetes, as unsuccessful transitioning can lead to increased hospitalizations (4). Studies in this area demonstrate clinical benefit, though participants willing to engage in additional transition-related care may be more motivated to access care in general (4, 5). There is similar interest in robust transition programs so that patients with Turner syndrome also receive high-quality medical care as adults, particularly given the evidence that they are often lost to follow-up as adults (6); this is described in more detail from a hypogonadism standpoint below. The general aspects of transition and adolescent medicine apply in the field of endocrinology, with additional considerations specific to each condition.

When considering the transition of children with metabolic bone disorders and structuring a robust strategy for management as adults, the heterogeneity of the disorders themselves becomes evident. This provides additional challenges in developing standard care plans. The relative paucity of guidelines for these conditions in childhood is only compounded by the lack of guidance for adult endocrinologists as well as other providers caring for these patients. Some parts of this discussion are specific applications of general considerations: genetic counseling, logistics of continuous follow-up, insurance coverage of medication and services available to children, safety of medications during pregnancy, etc. In addition to general considerations, there are particular emphases for the bone health of adults with underlying metabolic bone disorders; even more detailed aspects for specific disorders will be enumerated below.

The use of imaging to assess bone health over time is a critical element of appropriate transition. Multiple imaging modalities are available to evaluate bone density and bone composition. Quantitative computed tomography (QCT) assesses the lumbar spine and proximal femur, and peripheral QCT (pQCT) assesses the distal radius and tibia. These are useful research tools, particularly to evaluate differences between cortical and trabecular bone, but their use in clinical practice is more limited (7, 8). To date, dual X-ray absorptiometry (DXA) remains the standard clinic tool for evaluation of bone mineral content (BMC) and areal bone mineral density (aBMD) in the pediatric population (7). DXA can be used for densitometry of the spine as well as total body less head (TBLH) (7). While DXA is an established tool for vertebral fracture assessment in adults, there are emerging data for its utility in pediatric populations, particularly with the newer machines (9, 10).

A key aspect of transition is the differences in the evaluation of bone health in children and adults using DXA. First of all, the bone densities for children, young and middle-aged adults, and older adults are evaluated using different reference data. Children continue to accrue bone during their growth, whereas adults have already achieved peak bone mass. This highlights the value of Z-scores for children whose bone measurements are compared to peers with adjustments for sex and age. Because children may have different growth trajectories and bone density is underestimated in shorter children when relying on areal measurements obtained by DXA, BMD is ideally adjusted for height z-score^1^ (11, 12). Adults, on the other hand, have finished growth and bone accrual and have different fracture risk depending on age, sex, and estrogen status for women. For this reason, women and men < 50 years of age are compared to age and gender-matched references, and their DXA reports should use Z-scores similar to the assessment in children^2^ (13). T-scores, which are based on reference data derived from young Caucasian women, are recommended for menopausal women and men ≥ 50 years due to their correlation with fracture risk in this age group (13, 14). The quality of the images is also influenced by factors such as body habitus and positioning, both of which may affect the imaging of children and young adults with the metabolic bone disorders below; DXA results require careful interpretation and consideration of alternate sites of measurement.

The preferred sites for BMD measurement differ between children and adults. Recommended sites for DXA measurement in most children are TBLH and lumbar spine. The proximal femur is not preferred for most children due to variability during growth. Exceptions include for children with limited weight-bearing in the lower extremities who are at higher risk for impaired bone health at that site (when reference data are available), and for adolescents who are anticipated to require continued surveillance into adulthood for the purposes of comparison. If these preferred sites cannot be measured due to surgery/hardware/positioning, DXA may be measured at the 33% radius in children if reference data are available. Lateral distal femur is another alternative site which may be used in non-weight-bearing children, provided reference data are present (12). In adults, preferred sites for BMD assessment include the lumbar spine, total hip, and femoral neck (if skeletally mature). Forearm or 33% radius may be assessed in select circumstances. These alternatives may be necessary, for example, if the hip or spine cannot be measured due to hardware, recent surgery, or severe obesity; and in hyperparathyroidism where cortical bone is preferentially affected over trabecular bone (13).

An additional consideration in applying DXA measurements across the lifespan is the relationship of statistical scores and radiologic diagnoses. In children, the standard terminology is “low bone mineral mass” or “low bone mineral density” for BMD Z-scores less than or equal to -2 standard deviations (SD) (12). “Osteoporosis” is not diagnosed in children unless there are clinically significant fractures: criteria include either atraumatic vertebral compression fractures, or low bone mineral density plus ≥ two long bone fractures by age 10 or ≥ three long bone fractures by age 19 (12). In contrast, “osteoporosis” is an appropriate term for T-scores -2.5 SD or less on BMD measurements at the femoral neck even without fracture; this is applicable to postmenopausal women and men 50 years and older (13). In adults who have not yet aged into these criteria, Z-scores are preferred, with a Z-score of -2 SD or less designated as a BMD “below the expected range for age” (13). Notably, the standard statistical reports are similar between children and young adults.

As patients enter adulthood, the availability of matched peers for comparison of DXA data is problematic. Because the BMD of young adults with metabolic bone conditions is compromised throughout childhood, there may be deficits in bone accrual and peak bone mass. These deficits will be reflected in Z-score comparisons to age-matched standards in a healthy reference population. As a result, the use of BMD measurements to inform clinical questions, such as the risk of fracture or the effect of pharmacotherapy, is often unclear. The FRAX^®^ (Fracture Risk Assessment Tool) that incorporates these BMD data may have similar limitations in terms of assessing the fracture risk for younger adults (15) and adults with metabolic bone disorders not included in the calculator. Because BMD measurements obtained by DXA may differ between machines, the potential for systematic differences between two machines used for the same patient in a pediatric and then an adult center must also be considered (16). The distinction between pediatric and adult standards is evident in the review of current practices for the specific conditions and exposures described below, wherein different imaging strategies may be employed in pediatric and adult medical settings for the same patient.

Given what is known and not known about the management of adults with childhood metabolic bone disorders (both the medical standards themselves and the logistics of implementing them in a complex healthcare system), the purpose of this review paper is to compile some of the discussion around and evidence for best practices in transitioning and planning long-term care for adolescent patients with metabolic bone disorders. Representative primary bone disorders discussed in this review include hypophosphatasia, X-linked hypophosphatemic rickets, and osteogenesis imperfecta. Secondary bone disorders include considerations of bone mineral density and optimal bone health in relation to hypogonadism, eating disorders, and childhood cancer survivorship.

## 2 Primary metabolic bone disorders

### 2.1 Hypophosphatasia

Hypophosphatasia (HPP) is a genetically inherited condition attributed to a loss-of-function mutation in *TNSALP*, the gene that encodes tissue-nonspecific alkaline phosphatase (ALP) (17). The inheritance pattern can be either autosomal recessive or autosomal dominant (18). Population-level mutation analysis has been performed, but incidence and prevalence data are limited by the mild and often undetected cases (17, 19). Three-dimensional modeling has been used to explore the effect of specific pathogenic mutations on ALP activity as well as the association between the genetic changes and clinical phenotypes (20). Such knowledge has implications on genetic counseling for family planning in patients with HPP, including patients diagnosed as adults.

The manifestations of HPP in children cover a broad spectrum of severity but are also classified as discrete clinical subtypes (21). In the most severe forms of the perinatal lethal subtype, children who survive childbirth are born with skeletal hypomineralization and typically succumb to respiratory failure (19). The clinical presentation in infancy or childhood is associated with limb deformities, fractures, and limitations to mobility; on the other end of the spectrum are patients with primarily dental anomalies or with *in utero* bowing and shortening of limbs with apparent improvement after birth (19). Some patients have a phenotype limited to effects on dentition, including premature loss of deciduous teeth (17).

Targeted pharmacotherapy has historically been lacking for HPP. Whether it is a current or a past treatment for an adult patient with HPP, unnecessary calcium and vitamin D supplementation can lead to hypercalcemia and hyperphosphatemia, respectively (19). As a decoy of inorganic pyrophosphate, bisphosphonates have a theoretical risk of further suppression of ALP activity and, as modulators of bone resorption, have relatively little effect in a disorder of bone mineralization (19). More recently, enzyme replacement therapy with asfotase alfa has become available for patients with severe HPP and is currently approved for patients with perinatal, infantile, and juvenile-onset HPP (19). This therapy is associated with improvements in skeletal formation, growth, and symptoms of disability related to the disorder (22).

No standard guidelines exist for the transition of care in patients with HPP as they move through adolescence into adulthood. Of critical importance in the modern era is the provision of asfotase alfa to adults with HPP diagnosed in childhood. A recent non-randomized clinical study in fourteen such patients found significant improvement in six-minute walk test and timed up-and-go test in these patients, as well as patient-reported pain intensity and health survey items related to physical health (23). More data in this area may allow for the development of guidance to support adult endocrinologists in evaluating their patients with HPP for this medication.

In addition to demonstrating clinical benefit, trials that measure the effects of asfotase alfa in adults with HPP also highlight the clinical spectrum of symptoms and disease that must be monitored. As discussed below, complications of HPP develop over time. Therefore, young adults with the condition may sustain new fractures, musculoskeletal pain, or debility (24). This may be particularly difficult for these patients to navigate without the support of a multidisciplinary team.

BMD assessment in adults with HPP is useful for monitoring bone composition and provides some evidence of fracture risk. However, a cohort study at the University of Wuerzburg found that there were some limitations to DXA evaluation in these patients. For example, higher bone density measurements in these patients may indicate ectopic calcifications near the spine as well as qualitative defects in the structural integrity of the skeleton not captured by quantitative bone density measurements (25). In fact, the accumulation of inorganic pyrophosphate at these sites, while read as high-density, may signal an increased risk of pseudofracture (25). Data from this cohort also suggest that femoral measurements seem less affected by genotype and are thus a better site for osteoporosis evaluation. Adult endocrinologists who use serial DXA data to inform bone health and fracture risk over time must be aware of the technical considerations of DXA in patients with HPP.

The delayed diagnosis of HPP in adults precludes management by a pediatric endocrinologist or in a transition program. Some adults are diagnosed by biochemical evidence of low ALP activity, and others are diagnosed in the setting of recurrent fractures and weak bone structure that may have been documented previously in childhood (26). Metatarsal stress fractures and subtrochanteric femoral pseudofractures are common in this population (corroborated by a cohort of patients diagnosed with HPP as adults at the Mayo Clinic Rochester), and the presence of such fractures should raise suspicion for the diagnosis (26).

As introduced above, genetic counseling for adolescents with HPP as they enter adulthood is important. There is little information on the safety of asfotase alfa during pregnancy, which is pertinent for all patients started on this medication during child-bearing years^3^ (27). From a genetic standpoint, the different inheritance patterns and unclear genotype-phenotype relationship for all identified mutations in *TNSALP* make predictive counseling difficult. For example, a seemingly unaffected partner with a variant in *TNSALP* may be labeled as asymptomatic, whereas the mutation could have a dominant negative effect in the appropriate setting and even lead to future symptoms in the individual tested (28). Anticipatory guidance is also limited by the unknown future clinical features of the patient’s evolving HPP as well as the complexity of a phenotypic spectrum within the same family (28).

There are several tools available to patients and providers during the transition process to adulthood (**Table 1**). Soft Bones is a non-profit organization that offers resources and support to all stakeholders. From a research standpoint, the HPP Registry is a multinational registry of patients with HPP, including data on epidemiology, symptom burden, and other aspects of the condition. Note that Alexion Pharmaceuticals, the manufacturer of asfotase alfa, provides the infrastructure for the database. Non-profit organizations and registries like these provide support for patients and families throughout the lifespan, and they also benefit the medical team advocating for these patients.

**Table 1:** Organizational resources for patients with metabolic bone disorders.

	Advocacy Group	Patient Registry
**Hypophosphatasia**	Soft Bones, Inc.	Alexion
**X-linked hypophosphatemic rickets**	XLH Network	
**Osteogenesis imperfecta**	OI Foundation	OI Registry
**Klinefelter syndrome**	AXYS™: The Association for X and Y Chromosome Variations	
**Turner syndrome**	Turner Syndrome Foundation Turner Syndrome Society of the United States	Turner Syndrome Registry
**Survivors of eating disorders**	National Eating Disorder Association	
**Childhood cancer survivors**	Passport for Care^®^ with the Children’s Oncology Group	National Childhood Cancer Registry

### 2.2 X-linked hypophosphatemic rickets

X-linked hypophosphatemic rickets (XLH) is the most common cause of genetic hypophosphatemic rickets in childhood, with a reported prevalence of 1 in 20-60,000 people, and an incidence of 3.9 affected individuals per 100,000 live births (29, 30). XLH is caused by a loss-of-function mutation in *PHEX*, with a mechanism that is not entirely understood but ultimately leads to inappropriately increased activity of fibroblast growth factor-23 (FGF-23); as a consequence, urinary phosphorus wasting is promoted, and vitamin D activation is restricted (29).

Conventional therapy is comprised of phosphorus supplementation and the administration of calcitriol, since FGF-23 inhibits endogenous vitamin D activation to 1,25-dihydroxyvitamin D. More recently, burosumab, a monoclonal antibody targeted against FGF-23, has emerged as the primary pharmacotherapy for XLH and is FDA-approved in children and adults (31). There is limited data on the use of burosumab in asymptomatic adults, and there is also limited experience in continuing burosumab in adults who started the medication as children (32). This is problematic, because adults may have stopped medical therapy indefinitely, only to restart when symptoms develop (33, 34).

Burosumab has been shown to cause sustained improvements in mobility and pain in adults (31). Participants who were randomized to placebo during the first 24 weeks of a clinical study had significantly improved self-reported scores of stiffness and functional exercise capacity only after starting the 24 weeks of burosumab therapy. The mean additional six-minute walking distance after 24 weeks of burosumab was 23 meters (p<0.001) (31). As with asfotase alfa for HPP, the use of burosumab in adults with XLH has limited data in pregnant patients^4^ (35).

Adults with XLH may develop early osteoarthritis and enthesopathies (calcifications or bony proliferation at sites of ligament insertions) (36). A cohort of 52 young adults with XLH (mean age 41.8 years) in the French Reference Center for Genetic Bone Diseases showed a link between enthesopathy and decreased quality of life (36). These symptoms may lead to musculoskeletal pain and fatigue, findings that must be reviewed routinely in adults with XLH (36). While the pathophysiology of enthesopathy in patients with XLH is not fully known, it is thought to be related to chronic inflammation (37). It is unclear if past calcitriol and phosphorus treatment, which have significant benefit on the composition of the growing skeleton in children, alter the natural history of osteoarthritis and enthesopathies (36). This must especially be considered in adults who did not have access to burosumab therapy as children prior to their participation in adult endocrinology practices. Some young adults may bear the effects of unresolved symptoms of XLH as they transition out of pediatric care (38), in addition to further debility accrued during adulthood.

The effects of XLH on bone density appear to have a site-dependent effect (for example, axial *vs*. appendicular skeleton). Adults, but not children, with XLH had higher aBMD at the lumbar spine on DXA in one study (39), which may provide an additional challenge in selecting skeletal sites in these patients and interpreting their measurements over time. In an analysis of 34 patients (age 6 to 60 years) with XLH and an identified *PHEX* mutation, volumetric BMD was lower in cortical bone than trabecular bone on pQCT. The mean Z-score specific for age and sex was significantly lower in patients never treated with conventional therapy (-4.1) compared with patients currently on conventional therapy (-1.3), for cortical volumetric BMD measurements (40). Conventional therapy may improve BMD in these patients without normalizing it, and longitudinal studies will be needed to see the long-term effects of burosumab on BMD (40). As above, a distinction must be made between patients who started on burosumab soon after diagnosis and patients who were first treated with an extended course of conventional therapy or self-elected observation.

Cheung et al. surveyed children and adults with XLH; in analyzing the results of 295 respondents, they found that adolescents reported mood disturbances, and young adults expressed worries about the future (41). Pain seemed to be a more important symptom in adult respondents, and dental pathology was reported across the lifespan (41). It can be difficult for patients with XLH to find oral care professionals familiar with their specific dental needs, and this can add to the complexity of coordinating care for adults and developing a personalized medical care team (42). More general health concerns related to a higher prevalence of obesity and higher cardiovascular risk (additional evidence of the chronic inflammatory state of XLH) must also be considered in adulthood (37).

Genotype-phenotype correlation has been undertaken in different genetic populations with XLH (30, 43). There is some thought that truncating mutations of *PHEX* may predict more significant skeletal disease, but that even within the same family the inherited mutation has a spectrum of manifestations and severities (30, 43). Ultimately, there may be a genotype-phenotype correlation in some manifestations of XLH (30). More research is needed to provide a more accurate prediction of natural history in patients with particular mutations, information which would inform their own health risks and inherited risks to potential offspring.

The XLH network (see **Table 1**) is a non-profit organization that offers resources and support to all stakeholders, with the goal of early diagnosis and appropriate treatment for affected individuals and families. Building and studying an international registry for patients with XLH across the lifespan is an active area of research, and such a registry is actively being built, with its first constituents out of the United Kingdom (44).

### 2.3 Osteogenesis imperfecta

Osteogenesis imperfecta (OI) is a chronic, lifelong, hereditable, heterogenous collection of connective tissue diseases resulting in skeletal fragility and multisystem organ involvement; its primary manifestation is fracture. OI affects males and females in equal numbers. Generally, the incidence of cases recognizable at birth is estimated to be 1 in 10-20,000. More mild types may be recognized later in life and are felt to occur at a similar incidence (45, 46). Globally, 6.5 in 100,000 people have OI, translating to approximately 50,000 individuals in the United States^5^ (47). The vast majority of mutations in OI are autosomal dominant and affect type 1 collagen. However, increasingly, autosomal recessive forms and other mutations that do not directly affect type 1 collagen are being discovered (48).

Fragility fractures and low bone mass are the hallmarks of OI. Depending on the clinical severity of the condition (Sillence classification), these may be present *in utero*, at birth, or in childhood (49). Other musculoskeletal manifestations include short stature, limb and spine deformities, and restricted mobility; these may require surgeries, mobility assist devices, and/or adjustments of the physical environment. Over time, ocular, auditory, dental, cardiac, renal, metabolic, and pulmonary manifestations may also occur, increasing the burden of care for the person receiving healthcare. While the frequency of fractures decreases after puberty, involvement of other connective tissue systems tends to increase with age.

Musculoskeletal goals in treatment of OI include decreasing fracture incidence; improving pain; and promoting growth, mobility, and functional independence. To attain these goals requires a team of multidisciplinary specialists. This group would typically include a primary physician, dentists, audiologists, physical therapists, orthopedists, nutritionists, geneticists, endocrinologists, and social workers/care coordinators in childhood. As persons with OI age and manifestations move from the primarily musculoskeletal to involve other organ systems, other specialists might include pulmonologists, cardiologists, and ophthalmologists.

Pharmacotherapy for persons with OI is limited. Growth hormone is not considered standard of care at present but can be considered on an individual basis (50). In children and adolescents who have not yet reached their final adult height, bisphosphonate therapy is considered standard of care. Specifically, cyclical use of nitrogen-containing bisphosphonates (risedronate, alendronate, pamidronate, and zoledronate) is felt to improve bone mass, positively affect bone architecture, and possibly reduce fracture rate (51–53). Intravenous bisphosphonates appear to be superior to oral bisphosphonate with respect to improvements in BMD and reduction in fracture rates (54). Cyclical intravenous bisphosphonates also provide some pain relief (55). Similar beneficial effects are not found in adults; this is likely related to lower bone turnover rates in older persons. Denosumab, a human monoclonal antibody to receptor activator of nuclear factor kappa-B ligand (RANK- L), a cytokine mediating osteoclastogenesis and osteoclast survival, is currently being investigated as a therapeutic agent in pediatric OI. Currently, use of this in OI is considered experimental and has been reported in a case series of subjects with Type VI OI and a series of subjects with OI and osteoporosis (56, 57). It was generally well tolerated, but duration of effect on bone density was felt to be relatively short (months) and variable; hypercalciuria and hypercalcemia were observed in the subjects with Type VI OI. Gene-based therapies may lie in the future of OI therapeutics.

In adults with OI, there has been one double-blind clinical trial of use of teriparatide, which included subjects with a mean age of 41 years and an increased proportion of those with Type 1 OI. In the trial, subjects in the treatment arm had an increase in BMD at the lumbar spine and in the total hip measurements compared to the placebo group, and bone mineral turnover markers were increased in the treatment group when compared to placebo. There was no significant difference in fracture rates (58). An observational study by Gatti et al. also showed an increase in BMD after 18 months in subjects previously treated with neridronate (59). Most recently, sclerostin inhibitors, which affect the Wnt pathway to increase bone formation and, to a lesser extent, decrease bone resorption, are being studied in adults with OI (60). Finally, increased TGF-β activity has been noted in mouse models of OI and leads to lower bone mass and strength and increased fractures. Fresolimumab, an antibody that can silence TGF-β, has recently completed an adult phase 1 study establishing safety and dosing protocols for larger investigation (61).

Health-related quality of life (HR QoL) in affected adults has been studied. Individuals with OI experience varying degrees of physical limitations, which may be accompanied by pain and fatigue and can affect quality of life through alterations in social function, peer acceptance, and ability to perform activities of daily living. In a systematic review of literature discussing the psychosocial implications of OI for individuals, Tsimicalis et al. highlighted the need for caregivers, healthcare professionals, administrators, and policy-makers to understand the major concerns of persons with OI. They characterized their findings as encompassing several domains: intellectual feats, isolation and feeling different, fear of fractures, coping with challenges, adapting by learning new skills, and social relationships. They recommended a series of actionable steps, including improving upon the quality and direction of research in psychosocial and quality of life issues in OI, developing ways to address currently identified concerns, and providing preventative counseling/care for issues they may encounter over their lifespan. Use of information technology was seen as a particular opportunity to improve care (62).

An Osteogenesis Imperfecta Foundation (OIF) sponsored Patient-Centered Outcomes Research Institute (PCORI) study of adults (mostly North American based) with OI was performed to identify issues of specific HR QoL concerns for adults with OI and to inform future research efforts designed to improve quality of care for them (63). Totsi et al. noted a “higher prevalence of musculoskeletal and auditory problems and specific QoL concerns regarding the musculoskeletal, auditory, pulmonary, and endocrine systems” when compared to the general population. This finding expands the range of topics pursued in research and clinical settings. Additionally, the finding of the homogeneity of the respondents (female, middle aged, white, educated) highlighted an important gap in OI research, lack of diversity. For transition programs, this suggests that persons of minority status, male gender, and lower educational level may be at particular risk of not transitioning to adult care and may require redoubled individualized efforts and open communication.

In a study of HR QoL of Dutch adults with OI, a significantly lower reported QoL was noted when compared to the general population. The majority of respondents had type 1 OI, and more than half were male. Specifically, the adult OI cohort reported significantly decreased psychosocial and physical QoL across multiple domains and age groups, compared with the control group(s). Social function was mostly reduced in respondents younger than 25 years, but there were some differences for those older than 25 years when compared to the general population. The authors plan to develop interventions and then reassess for influence on QoL (64).

Bone density evaluation in OI is used for monitoring of disease progression and assessment of response to therapy with bisphosphonates (65). For individuals receiving bisphosphonate therapy, a baseline scan is performed and repeated at regular intervals (66). Changes in the DXA can be used to guide treatment with respect to dosing and interval (67). Importantly, the presence of metal implants skews the measurements obtained on DXA (68). Because intramedullary rodding is used to promote bone strength in patients with OI (69), serial DXA evaluation can be challenging; changes in measurements over time must take this variable into consideration. As above, differences exist between adult and pediatric populations with respect to reporting (T-score *vs*. Z-score) and primary sites (TBLH *vs*. femoral neck). In considering both the potential for metal implants and the changes in sites of measurement on DXA across the lifespan, alternative skeletal sites can be measured over time in these patients for consistent monitoring (70).

Given that this condition is often inherited in an autosomal dominant fashion, genetic counseling should be considered an essential component of care. Many individuals with mild OI will have an affected parent (up to 40%). Genetic testing of both child and parents is required to determine whether the OI is inherited from a parent carrying a mosaic mutation (occurs in up to 16% of new cases) and confers an increased risk of recurrence in subsequent offspring^6^ (71, 72).The risk to the siblings of the affected person is related to the carrier status of the parents. It is important to acknowledge that genotype-phenotype correlation in OI can have clinical variability even within family members carrying the same variant (46, 73). While genetic counseling often occurs when an individual is identified, for those who were diagnosed in infancy or childhood, genetic counseling should be readdressed to the adolescent or young adult. Optimal timing of this varies on an individual basis.

Review of specific transition programs for OI care can be seen in this report of a Shriner’s Children’s Hospital program for transition of care in pediatric bone conditions, including OI, which was conducted *via* survey of participants (74). A multidisciplinary committee oversees the program, whose focus includes not only the transition of the person receiving medical care to an adult provider, but also his/her/their education, employment, living arrangements, and social life. From the age of 14 years, a transition coordinator is incorporated into the care team, and an individualized plan is developed. Persons receiving care can also elect to attend camps or other social events where they can acquire life-skills independently from their family unit. At between 18 and 21 years, the individual is instructed to obtain a copy of their medical records, and at their last visit to the pediatric services, the individual is given a wallet sized card with contact information for the adult providers to contact the pediatric care team if needed.

Although a small sample size limits its power, themes here echo many other reviews of transition programs. Most participants identified strengths of the program, including a well-designed transition model, a monitoring system, and an inter-professional team working in conjunction with the medical organization. However, a change in personnel and organizational focus led to decreased effectiveness of the program. Furthermore, adult physicians felt that young adult OI persons receiving care “can be demanding and ill-prepared for the reality of the transition to the adult world.” Poor interface of electronic medical records (EMR) was also felt to be a barrier as well as lack of physicians with an interest in caring for young adults with OI. Similarly, those transitioning were dissatisfied with having to transition to an adult care hospital, resulting in a change from them using the system for preventative services to more acute care use. The authors cited a need for a strong skilled leader for the program, appropriate funding and resources, and incorporation of the family unit. Additionally, the authors noted opportunities in using information technologies and improving information sharing between pediatric and adult hospital systems.

Best practices in transition care for persons with OI is an area of current investigation, and there are some principles to keep in mind. OI-specific transfer tools may assist in the process of transition (75). Physician involvement will vary with the degree of severity of the condition, but both persons receiving care and the physicians need to be cognizant of possible complications and at-risk situations (e.g. complicated joint injuries in milder forms of OI). Continued attention to appropriate therapies in young adults may be limited by scheduling and medical insurance benefits. Loss of therapies can decrease functional ability and physical stamina. Platonic and sexual relationships are very important for young adults. Counseling related to these as well as future counseling related to childbearing need to be provided during the transition phase in a candid person-focused fashion (76). Tools for transition of care in OI can be found at the OI foundation website^7^ (77), which is also listed in **Table 1**.

## 3 Secondary metabolic bone disorders

### 3.1 Hypogonadism

Hypogonadism encompasses a large group of conditions, both congenital and acquired, that lead to low production of sex steroids and, in children, result in delayed or absent pubertal development. We will discuss bone issues and the transition of care in several causes of hypogonadism: delayed pubertal development, congenital hypogonadotropic hypogonadism as an example of hypogonadotropic hypogonadism, and Klinefelter syndrome and Turner syndrome as examples of hypergonadotropic hypogonadism/primary gonadal failure phenotypes with low bone mass. Discussions concerning hypogonadism related to functional amenorrhea in anorexia nervosa and in those receiving cancer treatments are discussed below. While there are also multiple conditions that result in hypogonadism in adults, we will limit our discussion to those affecting pediatric persons.

Bone mass attained early in life is perhaps the most important determinant of lifelong skeletal health. Growth in bone size and strength occurs in childhood and is complete by the third decade of life, making the accrual of peak bone mass largely a pediatric and young adult phenomenon. A 10% increase in peak bone mass gain can have significant influence on risk of osteoporosis later in life, and in one analysis, it postponed onset of osteoporosis by 13 years (78, 79). Suboptimal peak bone mass may be related not only to osteoporosis in later life but also to fractures in childhood and adolescence (80). Later timing of the adolescent growth spurt can affect bone mineral density of the total body, spine, femoral neck, and radius (81).

Estradiol, obtained from aromatization of testosterone in males, and by production from ovaries in females, is considered the central sex steroid regulating bone mass accrual to peak bone mass and skeletal maintenance throughout life (82). Testosterone action on the androgen receptor actively contributes to periosteal expansion of the male skeleton during pubertal development but is less important in trabecular and cortical bone development and acts on osteoblasts and osteocytes (83–85). Because sex steroids are important for bone health, hypogonadism leads to decreased bone mineral density during the period of time during which peak bone mass is accrued and can thus result in a lower total bone mass in adulthood if not recognized and corrected.

#### 3.1.1 Constitutional delay of growth and puberty

Delayed puberty is defined as pubertal onset occurring at an age of 2 or 2.5 SD later than the population mean. The traditional clinical endpoints for typical pubertal development are testicular volume of ≥ 4 mL by age 14 years in males and breast development (Sex Maturity Rating 2) by age 13 years in females. Pubertal tempo is also taken into account but more challenging to define; a recent Danish study has proposed a pubertal nomogram for males (86). The most common cause of pubertal delay is constitutional delay of growth and puberty (CDGP), previously known as constitutional delay of growth and development, which is more common in males than in females (87). This is considered an extreme variant of the normal timing and sequence of pubertal development.

Pubertal delay is generally associated with lower aBMD in bone lumbar spine and femoral neck in both sexes (88). Later age at menarche may be associated with the development of osteoporosis although few studies exist that describe findings in women with frankly delayed pubertal development (89–91). Some studies in women with delayed pubertal development do describe increased fracture rates: vertebral, forearm, and hip (92–94). Many BMD studies focused on males with CDGP have come to differing results. Finkelstein et al. and Bertonelloni et al. found reduced adult aBMD in males with constitutionally delayed puberty who did not receive any hormone replacement (95, 96). However, in Bertonelloni’s study and in a study by Yap et al., volumetric BMD (calculated) was not any different in CDGP than in controls (97). In a small cross-sectional study of males with CDGP using quantitative ultrasound to assess bone density, untreated subjects had decrease in BMD over the 12 month period of observation (98). Nevertheless, in a study of bone turnover markers in males with CDGP, values remained in the normal range for references based on pubertal stage (99). Another study compared bone turnover markers in children with CDGP, familial short stature, and typically developing boys and found a difference only in the urinary deoxypyridinoline levels (100). Moirera-Andres et al. opined that finding decreased prepubertal mineralization in children with CDGP might suggest an inherent predisposition to osteopenia, rather than a later function of sex steroid exposure delay (101).

Treatment of extreme CDGP in males has relied on provision of low doses of testosterone for a short period of time, referred to as “priming.” A small series of testosterone treatment in males with CDGP performed for 6 months showed significant increases in BMD and BMC over that time period (102). Another treatment recently studied, letrozole, showed promising results in a meta-analysis; its benefit in these patients is *via* inhibiting the conversion of testosterone to estradiol, thereby slowing bone maturation (103). At 6 months, subjects receiving letrozole showed higher increases in serum luteinizing hormone, follicle stimulating hormone, testosterone, and inhibin-B compared to children who have received testosterone treatment (103). Bone health was not evaluated. Longer term studies with letrozole are needed as its use has been shown to decrease bone density *via* its suppression of estradiol. Differing results are found concerning sex steroid use and BMD in CDGP (104). There is little literature on treatment of CDGP in females with respect to bone health outcomes. There is no indication for regularly scheduled DXA scans for this population. Transition care is important to ensure that puberty is complete and fertility potential realized.

#### 3.1.2 Congenital hypogonadotropic hypogonadism

Congenital hypogonadotropic hypogonadism (CHH) is a rare disorder that results from the failure of the normal episodic release of gonadotropin releasing hormone (GnRH), leading to delayed puberty and infertility. If this condition is accompanied by an absent sense of smell, it is termed Kallmann syndrome. CHH is characterized by rich genetic heterogeneity; to date, mutations in more than 30 genes have been implicated in its origins (105). The prevalence/incidence of this condition is unknown, but there appears to be a male predominance of cases. In infancy, males may present with micropenis and cryptorchidism because of lack of the sex steroid surge during the minipuberty of infancy.

The most common manifestation of CHH is pubertal delay. As in other conditions with low sex steroids, adults with CHH have low aBMD that is responsive to testosterone/gonadotropin therapy (106). In a cross-sectional study of men with CHH receiving testosterone and/or gonadotropin therapy, the subjects with CHH all had lower aBMD levels than controls at all relevant sites. The authors posit that perhaps the mode of delivery of the sex steroids (injected testosterone *vs* transdermal methods) or even the choice of testosterone replacement *vs*. human chorionic gonadotropin (HCG) hormone replacement may influence outcomes. Interestingly, the authors also note that earlier initiation of therapy with some sort of sex hormone replacement may provide some benefit (107). Other studies have suggested that there is a critical window during which initiation of sex steroid replacement may have the most beneficial effects on long-term bone health, underlining the need for timely diagnosis (108). Finally, for genes whose expression directly effects bone health in addition to their association with CHH (*FGF8*, *FGFR1*, and *SEMA3A*), mutations may have effects on bone that confound the reported effects of hypogonadism itself (105).

Counseling should include that adherence to sex steroid/gonadotropin therapy is not only important for maintenance of sexual function and fertility, but also bone health. Transition care in CHH follows the general principles previously outlined. There is a European group that specifically advocates for evaluation of patient readiness, communication between adult and pediatric providers, and ensuring initial adult consultation has been completed^8^ (109, 110).

#### 3.1.3 Klinefelter syndrome

Klinefelter syndrome (KS) is a relatively common, highly heterogenous genetic condition classically described as having a 47,XXY (or 48,XXXY, or 49,XXXXY) karyotype (mosaic is also possible), tall stature, small hyaline testes with inadequate testosterone production and oligospermia or azoospermia, and a particular neurocognitive phenotype (111). Its prevalence is approximately 1 in 500 to 1,000 males, with many diagnoses missed until adulthood, and even then, many diagnoses are missed altogether (112). The central physical finding is a small testicular volume. The genetic background for KS is based on sex chromosome non-disjunction, which leads to the presence of extra X chromosome(s). Mosaicism (mainly 46,XY/47,XXY) is present in around 10–20% of persons with KS. Usually, boys with KS enter puberty typically and testosterone rises in a physiological fashion, allowing epiphyseal closure and satisfactory development of secondary sexual characteristics; a small portion of boys may present with pubertal delay (113). However, over time, there are increased gonadotropin levels and decreased testosterone levels/action (and lower estradiol levels).

Although children with KS have been found to have lower bone density than their peers even in prepubertal stages (114), it is felt that the decreased testosterone in KS greatly contributes to the finding of low BMD. Fractures and osteoporosis are more common in KS men than in typical controls (115). A recent meta-analysis of studies which provided testosterone treatment to men with KS found that testosterone replacement resulted in increases in the lumbar spine (LS) measurement on DXA similar to men with functional hypogonadism receiving testosterone therapy (116). Other studies have highlighted the possible contributions of low 25-hydroxyvitamin D levels and low insulin like factor (INSL3) to the low bone density found in men with KS (112).

Treatment in KS of low bone density consists of testosterone, 25-hydroxyvitamin D, and calcium supplementation; osteoporosis is treated in the same way as for those without KS. Delivery method can significantly affect treatment, with those receiving transdermal testosterone more likely to continue medication (117). Counseling should include the importance of adherence to aforementioned therapies and the increased risks of fracture over the lifetime of a person with KS. There are few studies investigating transition care in KS. As in other conditions, Gies et al. propose a multidisciplinary team approach, including the urology, endocrinology, psychology, psychiatry, neurology, and genetics specialties (118). Focus for the young adult with KS might switch to concerns of hypoandrogenism and infertility and might not include long-term bone health risks (119). Support for self-advocacy for emerging adults with KS is summarized in **Table 1**.

#### 3.1.4 Turner syndrome

Turner syndrome (TS) is a chromosomal condition that affects phenotypic females who have one intact X chromosome and complete or partial absence of the second sex chromosome in association with one or more clinical manifestations. TS occurs in about every 50 per 100,000 women worldwide, with a median age at diagnosis of approximately 15 years (120). However, many women with Turner syndrome are not diagnosed until adulthood, and like KS, there are a great many likely missed diagnoses which constitute a milder phenotype (121, 122). The classical findings of TS include short stature, premature ovarian failure, congenital cardiac anomalies including changes to the aorta (and its valve), renal anomalies, increased risks of autoimmune conditions of which hypothyroidism and celiac disease are most common, hearing loss, and a specific neurocognitive phenotype (123). There is variability in the phenotype, and karyotype-phenotype correlation is challenging; some experts have suggested using a percentage of 45X cells present in peripheral leukocytes or other tissues to be able to better predict screening and counseling needs (124).

The skeletal fragility phenotype in TS is multifactorial. There is interplay between the underlying primary skeletal dysplasia; the presence of premature ovarian insufficiency; other co-morbid conditions often found in TS; and a propensity to falling from deficits in height, vision, and visual-spatial processing (125). Fracture ranks among substantial morbidities of TS, both osteoporotic fragility fractures in adulthood and non-osteoporotic fractures in childhood (126). Decreased BMC is noted in the absence of estrogen replacement therapies; 50-80% of women with TS not receiving estrogen have low BMC (127).

The mainstay of therapy for bone health in women with Turner syndrome and ovarian insufficiency remains estrogen replacement therapy. Timely initiation of therapy and its maintenance can improve and maintain BMD (128). Guidelines do not specify which route is superior, but transdermal routes of estradiol replacement are preferred (123). Women with TS who are diagnosed in childhood may also receive growth hormone (GH) therapy. In one retrospective study of 28 women with TS (half received GH and half did not), GH treatment was associated with increased height, larger sized bones, and one measure of increased bone strength. No differences were found in aBMD, high-resolution pQCT-derived parameters, or another measure of bone strength (129). DXA is recommended to be performed at the initiation of adult hormone replacement therapy (not during pubertal induction) and every 5 years thereafter. It is important to note that short stature of women with TS may affect interpretation of DXA, and this should be conveyed to the reader of the study (123).

Transition care of women with TS has been studied, and multiple resources are provided in **Table 1** (130). Recommendations include a staged transition with increasing involvement of adult providers, recognition of later complications including osteoporosis and dysmetabolic syndrome, and emphasis on counseling concerning maintenance of healthy lifestyle and physical activity. Use of a combination of general transition readiness tools (TRAQ)^9^ (131) and specific TS transition tools (e.g. TS Pediatric to Adult Care Transition Toolkit and the Turner Syndrome Transition Passport^10^ available from the Pediatric Endocrine Society) is recommended (132). Key content areas to be addressed during this period include estrogen therapy and reproductive issues; cardiovascular health; lifestyle requirements; and psychosocial, educational, and vocational issues to ensure increased HR QoL. A brief period of co-management between pediatric and adult providers may be of benefit (123).

### 3.2 Eating disorders

Eating disorders are a diverse group of illnesses that include anorexia nervosa, bulimia nervosa, and avoidant restrictive food intake disorder. These disorders often manifest in adolescents, affecting 0.2 -15% of the teen population (133). The nutritional and endocrinologic effects of eating disorders can have profound effects on BMD. As discussed previously, in the adolescent population this effect is even more pronounced, as up to 90% of BMD is developed during the teen years (134). Consequently, the lifetime risk of fracture for those with anorexia nervosa is 2 to 7-fold higher than the general population (135–137). In contrast to other medical complications of eating disorders, the effects on BMD may not improve with weight restoration alone (134, 138, 139). It is also important to note that low weight may not be a necessary component of an eating disorder diagnosis, as patients with extreme weight loss or weight fluctuations may also experience malnutrition and BMD loss (138).

The etiology of low BMD in patients with eating disorders is multifactorial. The most commonly cited risk of BMD loss is the downregulation of the hypothalamic-pituitary-gonadal axis, often clinically evidenced in females by amenorrhea (134, 138, 140, 141). This downregulation, which results in low estrogen and testosterone, directly and indirectly increases osteoclastic activity (134). Additionally, up to one third of patients with anorexia nervosa show increased cortisol production, which leads to increased bone resorption and decreased osteoblastic activity (138). Many patients with eating disorders also exhibit low levels of insulin-like growth factor (IGF), low levels of IGF carrier proteins, and resistance to growth hormone activity, resulting in further bone loss (140, 142). For many patients with eating disorders, low lean body mass diminishes the anabolic action of muscle on bone growth, resulting in decreased bone formation (134). Finally, some aspects of treatment, such as selective serotonin reuptake inhibitor use, cause additional negative impact on BMD (143).

Current treatment strategies to address the risk of BMD loss in eating disorders are based on the severity of osteopenia or osteoporosis at the time of diagnosis. Weight restoration is the mainstay of treatment (with resumption of menses in women) but has been noted to be insufficient to fully restore BMD in some cases and does not fully eliminate future fracture risk (139, 144–148). In addition to sufficient caloric intake, adequate vitamin D and calcium intake should also be assured to optimize potential outcomes (134, 149). For those who would benefit from additional intervention, transdermal estrogen can provide some improvement in BMD (150). Oral bisphosphonates, as with osteoporosis from other causes, can improve BMD at 12 months (151, 152). Teriparatide also provides significant improvement in BMD but has limited use in adolescents with open epiphyses (153).

For patients with a history of eating disorder and bone density loss who are transitioning into adult care, the goals of care should consider the patient’s current status in eating disorder recovery (see **Table 1** for patient-facing support). For patients who have a history of eating disorder with osteopenia or osteoporosis, BMD loss may persist despite weight improvement. Thus, follow-up DXA every 1-2 years until BMD is normalized is a reasonable approach. For patients who have not yet restored to goal weight, the primary initial endpoint should be weight restoration. Until weight is restored, patients should continue care with a multidisciplinary team versed in eating disorder care to address the nutritional, emotional, and psychiatric elements of the condition. Additionally, patients may require agents such as estrogen and/or bisphosphonates until cycles are restored and weight is appropriate. Patients may also become candidates for additional medications such as teriparatide as they age. There are no definitive guidelines for which therapeutic agents to employ, and these may be tailored individually to each patient based on severity of disease, medication compliance, and risk/benefit profile. For all patients with adolescent onset of eating disorder, it is important to consider that these patients are at higher risk for relapse than other patients, up to 41% (154). Physicians should carefully follow weight trends and address potential for relapse with consistent weight loss, ideally before the body mass index (BMI) is low.

### 3.3 Cancer survivorship

From 2015-2019, cancer in those 19 years or younger in the United States was estimated to occur at a rate of 201 and 186 per 1 million for males and females, respectively (155). With improvements in therapy, the survival rate in childhood cancer now approximates 85% in the United States^11^ (155). Consequently, there has been an increased focus on long-term health consequences in this population, including bone health. Despite this, there are many unanswered questions about the full effects of cancer and cancer therapy on future bone health.

Conventionally, low BMD for age in pediatrics is defined as a BMD Z-score ≤ - 2 (156). In the childhood cancer population, many studies in the medical literature designate lower BMDs as very low BMD (Z-score ≤ -2) and low BMD or osteopenia (Z-score between -2 and -1) (157–160). The Children’s Oncology Group designates reduced BMD as a Z-score < - 2 if age < 20 years and a T-score < -1 if age ≥ 20 year^12^ (161). These distinctions are made to identify those at future risk of osteoporosis and fragility fractures (157). Childhood cancer survivors with BMD Z-scores between

-1 and -2 (even if considered within normal limits for age) may fail to reach peak bone mass, and in conjunction with their risk for earlier frailty, increase the risk for fragility fractures at an earlier age than their peers (162).

Children with cancer may develop suboptimal bone health from several mechanisms: the cancer itself (leukemic or primary tumor/metastatic infiltration of bone), therapies (radiation, glucocorticoids), or other disease resulting from therapy (growth hormone deficiency, hypogonadism), weight loss/lower lean muscle mass, and decreased physical activity (157–160, 162, 163). It should be noted that patients diagnosed with growth hormone deficiency may receive delayed treatment due to the need to wait a year until after cancer therapy is complete prior to start of therapy (164). This may also delay treatment of concurrent hypogonadism due to wish to preserve open epiphyses in growth hormone-deficient patients not yet on GH, and as mentioned above, timing of puberty or pubertal hormone replacement may affect overall peak bone mass.

Children with acute lymphoblastic leukemia (ALL) are especially at risk for low BMD (157, 163, 165). Other high-risk groups include recipients of hematopoietic stem cell transplant (HSCT) conditioned with total body irradiation (TBI) and/or treated with glucocorticoids for subsequent graft versus host disease, and brain tumor patients receiving cranial radiation therapy (CRT) or craniospinal radiation therapy (CSI) (157, 163, 165).

In particular, children with ALL have decreased lumbar spine BMD (LSBMD) (average Z-score < 0) at diagnosis (166–169) and a higher prevalence of very low LSBMD for age (Z-score ≤ -2) at presentation, with estimates ranging from 21% to 27% (167, 170). Since the lumbar spine mainly consists of trabecular bone, which is more metabolically active than cortical bone, changes in lumbar spine can be seen earlier than at other sites (167). Lower LSBMD at presentation is associated with higher risk for vertebral fracture, both at diagnosis and during therapy (166, 168, 170, 171). Vertebral fracture incidence at ALL diagnosis has been reported to be as high as 16% (166) with 39% asymptomatic (169). Low LSBMD is also associated with nonvertebral fractures (168, 171). One prospective study showed an 89% increased risk for vertebral fracture and a 70% increased risk for non-vertebral fracture for every 1 SD decrease in LSBMD at presentation (171). This reduction in LSBMD at diagnosis is attributed to multiple factors, including bone marrow expansion by leukemic infiltration, cytokines secreted by leukemic cells increasing osteoclast resorption, and lower lean muscle mass (165, 167, 169, 170, 172).

The extent of recovery of BMD and vertebral fracture following cessation of therapy is uncertain, particularly following leukemia treatment protocols. While a few prospective studies evaluated BMD during and just after therapy in ALL (167–169, 171), information regarding BMD and fracture trends in the immediate aftermath is mainly derived from the Canadian Steroid Associated Osteoporosis in the Pediatric Population (STOPP) consortium (169, 171). In a prospective study following children with ALL six years past diagnosis, there was a cumulative incidence of vertebral fractures of 36%, with vertebral fractures most likely to occur within the first two years of therapy (71%), and one of the strongest predictors of both vertebral and non-vertebral fractures was a vertebral fracture at diagnosis (171). 84% of those with a vertebral fracture at presentation had another vertebral fracture in the next two years (171). Of those experiencing a vertebral fracture, 77% underwent complete reshaping by the end of follow-up (171). Predictors of incomplete recovery were severity of the vertebral fracture and older age (171). Therefore, it is important to identify children most at risk for incomplete resolution of bone deficits to determine if early intervention can prevent persistent morbidity.

In 2021, the International Late Effects of Childhood Cancer Guideline Harmonization Group (IGHG) published recommendations for bone health surveillance based on available evidence, other clinical guidelines, and expert opinion, while highlighting gaps in knowledge (157). They differ from the Children’s Oncology Group namely in offering no recommendations regarding BMD surveillance for patients with a history of glucocorticoid exposure or HSCT in the absence of TBI, finding no significant association between low BMD and methotrexate exposure, and suggesting BMD surveillance based on specific types of radiation exposure (157).

Regarding therapy exposures placing children most at risk for persistent low BMD, the IGHG found that available data most strongly support an association with low or very low BMD with CRT, CSI, TBI, and pelvic/abdominal radiation (presumably due to associated hypogonadism) (157, 158, 160, 173–175). Disorders associated with low or very low BMD include hypogonadism, growth hormone deficiency, and 25-hydroxyvitamin D < 20 ng/mL (157). Patient factors include male gender, Caucasian race, low BMI, sedentary lifestyle, and history of smoking (157). Studies on fractures are limited; based on available literature, only male sex and higher dose glucocorticoids were associated with fractures (157).

The IGHG strongly recommends BMD surveillance for survivors with a history of CRT or CSI at 2-5 years post-completion of therapy and if normal, again at age 25 years, when peak BMD has presumably been reached (157). Those with hypogonadism and/or growth hormone deficiency should be screened as per standard of care (157). Screening BMD should also be considered for those exposed to TBI (157). DXA of the lumbar spine and total body less head is preferred over QCT for surveillance (DXA uses lower radiation and is more widely available) and although total hip DXA is not helpful in children, the authors advised assessment in adolescents who will be surveilled into adulthood (157).

The IGHG guidelines acknowledge that there is currently no data regarding early medical intervention (such as bisphosphonate use) in this population preventing future fractures, even if low BMD is detected (157). However, identifying low BMD allows promotion of nutritional and lifestyle guidance regarding calcium and vitamin D intake and physical activity, and may prompt evaluation for endocrine deficiencies which can be treated.

A retrospective, cross-sectional study from St Jude LIFE (involving periodic surveys accompanied by prospective in-person health screenings of St Jude cancer survivors) provided some reassurance regarding long-term bone health following childhood ALL (159). At a median age of 31 years, only 5.7% of survivors had a BMD z-score ≤ -2, while 23.8% had a BMD score between -1 and -2 (159). Of those with more than one BMD (median time between the tests 8.5 years), 92% improved BMD categories or remained stable (159).

For the overall cancer survivor population, a retrospective single center Dutch cohort study including various childhood cancer diagnoses identified a below-average total body (Z-score -0.55) and lumbar spine BMD (Z-score -0.30) in adult childhood cancer survivors (median age 24.5 years, median 16.7 years since diagnosis) (158). 45% had at least one BMD Z-score < -1.0 (158). However, the risk of future fractures is not well-established. The Childhood Cancer Survivorship Study (CCSS), a retrospective multi-site cohort study, revealed that at a median age of 36 years, 34.8% of survivors compared to 38.9% of siblings reported a fracture, most commonly an upper extremity fracture (176). Thus, survivors were no more likely to experience fracture compared to siblings, with male survivors slightly less likely to report fracture compared to siblings (176). When stratified by cancer diagnosis, results were similar, even in the leukemia survivors (176). The major limitation to these results was that fracture history was based on self-report through survey (176).

The literature is sparse regarding optimal interventions and timing of intervention, particularly for ALL patients, given that current data suggest that BMD improves with time (157, 159, 177), and the STOPP consortium suggests that the majority will develop vertebral reshaping without intervention (171). However, 23% did not experience full resolution, including those with more severe fractures and presenting at older age, and some patients went on to receive bisphosphonate therapy and were not further studied (171).

At present, available guidelines suggest that all at-risk survivors be counseled on lifestyle management to promote bone health, including physical activity and weight-bearing exercise, fall prevention, dietary daily intake of vitamin D of ≥ 400 international units and daily elemental calcium ≥ 500 mg, with supplementation suggested for 25-hydroxyvitamin D < 20 ng/mL, and nutritional supplements for underweight survivors (157, 178, 179). Of note, a double-blind randomized placebo-controlled trial of lifestyle counseling for bone health (including recommending optimal dietary intake of calcium and vitamin D) plus calcium and vitamin D supplementation *vs*. counseling and placebo demonstrated no additional benefit of supplementation beyond nutritional counseling (180).

IGHG recommendations for management of abnormal BMD or low-trauma fracture include referral to a bone specialist if BMD Z-score ≤ -2. In survivors with BMD Z-score between -1 and -2, evaluate for endocrine dysfunction which may cause low BMD and refer for management if appropriate, with repeat DXA in two years (157).

Due to the overall risk for chronic disease from previous exposures, there has been a lot of interest in appropriate transition of care of this complex patient population (see **Table 1**). Several models have evolved, including transitioning from pediatric to adult oncology, transitioning to primary care after communication with the oncologist, with subsequent referral to appropriate subspecialists, and use of survivorship clinics (either with shared care with local primary care providers or as the main provider of care) with referral to subspecialists as needed (181). However, regarding bone health specifically, as outlined by IGHG guidelines (157), surveillance depends on specific cancer history and exposures, and more evidence is needed to guide the optimal management of low BMD in adults. Ideally, an adult endocrinologist experienced in the metabolic bone effects of cancer-related exposures is part of the multidisciplinary team managing patients who have transitioned to adult care.

## 4 Conclusion

The idea of health transition for adolescents entering adulthood is not a new or unfamiliar concept, and most medical disciplines have patients with chronic medical conditions who have benefited greatly from robust transition programs (e.g. cystic fibrosis, type 1 diabetes mellitus, congenital heart disease). In fact, “transition to adult care” is a readily accessible Medical Subject Headings (MeSH) term in the PubMed database, reinforcing the importance of this topic in research as much as in clinical practice. As the field grows, so will the need for infrastructure; important elements include clinic space and time for the medical care, pediatric and adult providers well-versed in the needs of these emerging adults, nurses and other medical team members comfortable engaging this population from both a pediatric and adult standpoint, social workers and office staff allotted the time to coordinate the logistically complex medical care of these patients, etc. Telehealth may allow for more equitable care in terms of the barriers to in-person evaluation, such as travel, cost, and mobility (182). However, careful attention is still necessary to avoid additional barriers to equitable care using this tool, i.e. patient access to technology and wireless services, technical support in a patient’s own language, insurance coverage, and unbiased provision of opportunities for telehealth to all patients. Utilization of this resource as an appropriate healthcare setting will be an important element of future guidelines for the transition of patients with metabolic bone disorders.

Just as important as the infrastructure are the medical practices: the evidence-based approaches to transition in terms of medical decision-making and tactics. For the metabolic bone disorders reviewed here, the evidence available must be considered to guide surveillance and management strategies, applied to each patient’s particular experience. The extended lifespan and potential for improved QoL for patients with these conditions has allowed for the acquisition of long-term data to advance knowledge and understanding that can be applied to transition. One goal of continued research in areas that impact these populations is the opportunity to close some of the gaps that remain.

As discussed above, the age-based standards in measuring and reporting BMD measurements on DXA bring challenges to the study of outcomes over time. The osteoporosis-defining criteria differ in populations of different ages. Furthermore, the skeletal sites evaluated in children (TBLH and lumbar spine) are typically replaced with measurements of hip, femoral neck, and other sites for adults. To add to the complexity, the reproducibility and serial comparison of measurements on DXA is affected by bone accrual (which may be compromised in these patients) and implantation of hardware. If the adult provider involved in the transition process must use a different DXA scanner than the one used in a patient’s pediatric care, the accurate comparison of all DXA measurements directly over time is compromised. These aspects of densitometry must be thoughtfully incorporated into the surveillance strategy for each patient, with additional consideration to the underlying bone condition and its additional impact on BMD measurements.

Several themes arise from the metabolic bone conditions reviewed here. First, outcome measures, such as fracture risk in survivors of childhood cancer and eating disorders, are largely dependent on the standard of care for the cohorts followed. As higher success rates of less aggressive protocols for cancer treatment are developed, the outcomes in children currently undergoing these newer treatments may differ from those seen in previous studies of historical cohorts. Similarly, as primary care and specialty providers become more aware of the prevalence of eating disorders and develop a lower index of suspicion for evaluation and referral to eating disorder centers, the improved therapy may have an impact on the low BMD that stems from inadequate treatment of the underlying condition. As new modalities for estrogen and testosterone replacement become available, patients treated with the various formulations may have different risks of low BMD, as well. Data on transitioned populations may lag behind the active management of children and young adults entering the transition phase of their healthcare, and this perspective is important when reviewing the literature and thoughtfully applying it prospectively. Surveillance guidelines must be adapted with this constant flux of clinical practice in mind.

In addition to changes in exposure, changes in treatment for metabolic bone disorders also leave a natural lag between long-term data on adult populations and application to patients currently in the transition process. As detailed above, the benefits of asfotase alfa and burosumab in patients with HPP and XLH, respectively, will likely change the landscape of outcome data in these cohorts of patients followed over the coming years; in the same way, QoL data for these populations are likely to reflect the pronounced morbidity and fragility of conventional (or lack of) therapy less and less over time. While the associated complications of metabolic bone disorders as described above must still be considered, the relative amount of time that adult providers spend managing these complications may change over time. However, adult providers have the increasing responsibility of considering the indications for disease-specific medications as patients enter adulthood, and how cost and insurance coverage of these medications may change. In addition, access to health insurance during this vulnerable transition period can be difficult, resulting in lapses in coverage for medical services and medications that add to the complexity of coordinating care (1).

As the world of personalized medicine based on genetic data becomes more available and the variability in genotypes and genotype-relationships of genetic disorders affecting bone health becomes more characterized, the applications to transition medicine will be rich. Both a patient’s expectations for his/her/their future clinical course and guidance for hereditability and family planning will become more informed. While these predictive data points will not replace clinical observation and frequent monitoring, this information will empower patients with metabolic bone disease to face the future with more clarity than past generations.

While many other themes and patterns can be drawn from the previous discussion of metabolic bone disorders, a final consideration is two significant gaps. There is a gap between the robust data on outcomes in these populations and the limited number of guidelines synthesizing these data into actionable recommendations for adult providers and patients as they make clinical decisions together. There is also a literature gap between the available clinical recommendations (such as in XLH) and the experiences of persons who are managed in a transition clinic that incorporates these recommendations. For the first gap, review articles like this one are intended to consolidate information and resources but are not a replacement for guidelines developed by taskforces and committees of appropriate multidisciplinary stakeholders. For the second gap, the work in OI stands as an example of the strong link between clinical data, guidelines, and outcomes of transition clinics that is a meaningful goal for providers who have expertise in other bone mineral disorders.

The complexity of the data presented also validates the multiple specialties and team members whose input is needed in managing the care of and providing transition for these patients. For example, in a bone clinic where participants may have a diagnosis of an eating disorder that contributed to low BMD, an eating disorder specialist should be available throughout the lifespan to provide continued support for the underlying condition. Such multidisciplinary teams may be difficult as patients move away from pediatric healthcare systems that often collaborate in this way.

At the intersection of two exciting fields, transition to adult care and management of metabolic bone disorders are areas of medicine with unanswered questions but also many significant opportunities. While not historically at the forefront of transition medicine, metabolic bone disorders require careful consideration of the underlying genetic changes, pathophysiology, and exposures that drive them as well as reported adult outcomes and experiences. Children with these chronic conditions should have an intentional, strategic progression through adolescence into adulthood so that their medical care remains consistent and meets their specific needs.

## Author contributions

JR and AD-T contributed to the conception of the review. JR, MB, CY, and AD-T wrote sections of the manuscript. All authors contributed to manuscript revision, read, and approved the submitted version.

## Footnotes

1
https://iscd.org/learn/official-positions/pediatric-positions/ —Access date February 22, 2023

2
https://iscd.org/learn/official-positions/adult-positions/ —Access date February 22, 2023

3
https://www.accessdata.fda.gov/spl/data/6bb80772-f21f-4e48-b34c-ea66b6b15ccf/6bb80772-f21f-4e48-b34c-ea66b6b15ccf.xml —Access date December 19, 2022

4
https://www.accessdata.fda.gov/spl/data/81078e03-384d-40f9-a5e9-9b8b49163a32/81078e03-384d-40f9-a5e9-9b8b49163a32.xml —Access date December 19, 2022

5
https://oif.org/informationcenter/about-oi/oi-fast-facts/ —Access date December 15, 2022

6
https://www.ncbi.nlm.nih.gov/books/NBK1295/#oi.Genetic_Counseling —Access date December 15, 2022

7
https://oif.org/ —Access date December 15, 2022

8
http://www.gnrhnetwork.eu/ —Access date December 15, 2022

9
https://www.rheumatology.org/Portals/0/Files/Transition-Readiness-Assessment-Questionnaire.pdf —Access date December 15, 2022

10
https://pedsendo.org/wp-content/uploads/2020/06/Transition-Passport-Turner-Syndrome.pdf —Access date December 15, 2022

11
https://nccrexplorer.ccdi.cancer.gov/ —Access date January 3, 2023

12
http://www.survivorshipguidelines.org/ —Access date December 15, 2022
